# Effect of Nanoparticle Morphology on Pre-Breakdown and Breakdown Properties of Insulating Oil-Based Nanofluids

**DOI:** 10.3390/nano8070476

**Published:** 2018-06-28

**Authors:** Yuzhen Lv, Yang Ge, Zhen Sun, Qian Sun, Meng Huang, Chengrong Li, Bo Qi, Jinsha Yuan, Zhaoliang Xing

**Affiliations:** 1 State Key Laboratory of Alternate Electrical Power System with Renewable Energy Sources, North China Electric Power University, Beijing 102206, China; yuzhenlv@163.com (Y.L.); huang_m2011@163.com (M.H.); lcr@ncepu.edu.cn (C.L.); lqicb@163.com (B.Q.); 2 School of Energy, Power and Mechanical Engineering, North China Electric Power University, Beijing 102206, China; sunzh@ncepu.edu.cn (Z.S.); 15652912576@163.com (Q.S.); 3 School of Electrical Engineering, North China Electric Power University, Baoding 071003, China; yuanjinsha@126.com; 4 State Key Laboratory of Transducer Technology, Global Energy Interconnection Research Institute Co. Ltd., Beijing 102209, China; xingzhaoliang007@163.com

**Keywords:** nanorod, insulating oil, breakdown strength, streamer propagation, electric field

## Abstract

Nanoparticles currently in use are challenged in further improving the dielectric strength of insulating oil. There is a great need for a new type of nanoparticle to promote the application of insulating oil-based nanofluids in electric industries. This paper experimentally investigates the effect of nanoparticle morphology on pre-breakdown and breakdown properties of insulating oil-based nanofluids. The positive impulse breakdown voltage of insulating oil can be significantly increased by up to 55.5% by the presence of TiO_2_ nanorods, up to 1.23 times that of TiO_2_ nanospheres. Pre-breakdown streamer propagation characteristics reveal that streamer discharge channels turn into a bush-like shape with much denser and shorter branches in the nanofluid with TiO_2_ nanorods. Moreover, the propagation velocity of streamers is dramatically decreased to 34.7% of that in the insulating oil. The greater improvement of nanorods on the breakdown property can be attributed to the lower distortion of the electric field. Thus, when compared with nanospheres, pre-breakdown streamer propagation of nanofluid is much more suppressed with the addition of nanorods, resulting in a greater breakdown voltage.

## 1. Introduction

Nanoparticles have shown a promising prospect in improving the electric performance of dielectric materials [1,2]. The insulating oil-based nanofluids exhibit great potential to address the strong demands for power equipment with large capacity, high dielectric strength and small volume in an ultra-high voltage power grid [3,4]. The dielectric strength of insulating oils is closely related to the breakdown event, which is caused by the initiation and propagation of charged gaseous channels called “streamers” at the pre-breakdown stage [5]. Previous experimental evidence for insulating oils has shown that positive streamers emanating from the positive electrode tend to initiate at lower applied voltages and propagate faster and further than negative ones. As a result, impulse pre-breakdown streamers and breakdown under positive polarity constitute a great risk to dielectric strength in power equipment [6,7].

Extensive research has been conducted into dispersing nanoparticles into the oil to improve the dielectric strength of the insulating oil [8,9,10,11]. The lightning impulse breakdown voltage of insulating oil can be improved by 82.6% with the addition of Fe_3_O_4_ nanoparticles [8]. However, this conductive nanoparticle may increase the electrical conductivity of the insulating liquid and be influenced by a magnetic field which restricts its practical application [12]. In this case, insulating and semi-conductive nanoparticles (e.g., Al_2_O_3_ and TiO_2_) with lower electrical conductivity are widely used to improve the dielectric strength of the insulating oil. However, the improvement of positive impulse breakdown voltage by these nanoparticles is generally around 30% under optimum concentrations [13,14,15,16,17]. There is still a bottleneck in further increasing the dielectric strength of nanofluids modified by semiconducting nanoparticles. To date, only spherical nanoparticles have been used to modify the dielectric strength of insulating oil. Therefore, there is a necessity to investigate the effect of the microscopic structure of nanoparticles on the dielectric strength of insulating oil. The nanoparticle morphology may shed light on the enhancement of the dielectric strength of insulating oil-based nanofluid.

In this work, the effect of the TiO_2_ nanoparticle morphology on the dielectric strength of insulating oil-based nanofluids is investigated. TiO_2_ nanospheres and nanorods with a similar diameter were prepared by the solvothermal method and used to synthesize insulating oil-based nanofluids. The dielectric strengths of insulating oil and nanofluids with the same nanoparticle concentration were measured. To reveal the breakdown process, the propagation characteristics of pre-breakdown streamers were studied with the help of the schlieren technique. Moreover, the effect mechanism of nanoparticle morphology on the improvement of dielectric strength of insulating oil is proposed.

## 2. Experiment

### 2.1. Materials

TiO_2_ nanospheres and nanorods modified by oleic acid were synthesized by the solvothermal method in our lab [18]. Naphthenic transformer oil (25# Karamay) was used as the insulating oil, which was filtered to remove impurities and meet the demand for clean oil defined by CIGRE (International Council on Large Electric Systems) working group 12.17 [19].

### 2.2. Preparation of Insulating Oil-Based Nanofluids

TiO_2_ nanofluids (NFs) were prepared by dispersing TiO_2_ nanoparticles into the insulating oil with a concentration of 0.075 vol. % under stirring and ultrasonic treatment. The insulating oil and nanofluids were degassed at less than 1 kPa for 24 h before testing, and the moisture content of each sample was around 10 ppm.

### 2.3. Characterization and Measurement

**Figure 1:**
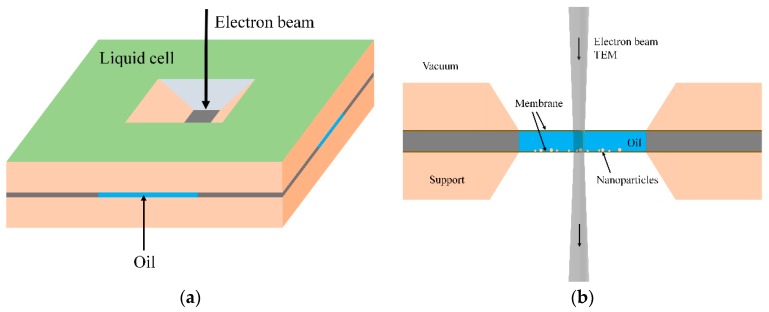
(**a**) Schematic of a liquid cell and (**b**) configuration for electron microscopy in oil.

The morphology of nanoparticle powders was observed by transmission electron microscope (TEM, JEM-2100, JEOL Ltd., Tokyo, Japan). However, limited by the vacuum environment of TEM, the morphology of nanoparticles in nanofluids cannot be observed directly. So, the liquid cell was applied to characterize the morphology of nanoparticles in nanofluids, as shown in Figure 1 [20,21]. The liquid cell was made from two vacuum-tight electron transparent membranes with a controlled separation of about 100 nm to 1 μm. Thin SiN films were used as membranes. The cell was filled with the oil. The electron beam was passed through the membranes and oil to allow recording of images [21].

**Figure 2:**
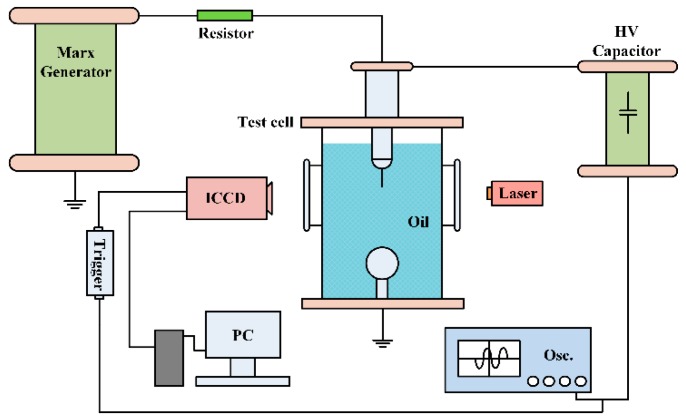
Schematic of experimental setup for breakdown and pre-breakdown measurement.

The breakdown and pre-breakdown properties were investigated by the experimental setup using schlieren technique, as shown in Figure 2 [22]. The breakdown property was measured according to the standard procedures for testing lightning impulse breakdown voltages (IEC60897-1987). A needle-sphere electrode system, with a high voltage tungsten needle (tip radius of 35 ± 5 μm) opposing a grounded sphere, was located within a test cell made of transparent Perspex sheet, which facilitates the streamer observation. An Intensified Charge Coupled Device (ICCD) camera was used to capture streamer propagation images, which were performed with an electrode gap of 40 mm under peak voltage of 75 kV. The camera works with the help of the laser to meet the need of the schlieren technique. A trigger unit was used to synchronously trigger the impulse generator and camera.

## 3. Results and Discussion

### 3.1. Nanoparticle Morphology

**Figure 3:**
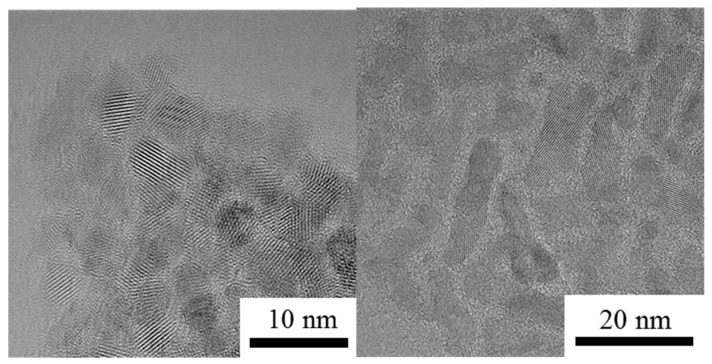
HRTEM (High Resolution Transmission Electron Microscope) images for TiO_2_ nanospheres and nanorods.

**Figure 4:**
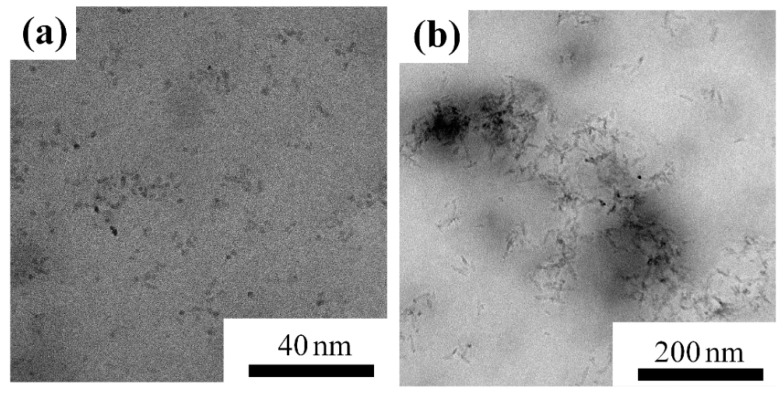
Transmission electron microscope (TEM) images for (**a**) TiO_2_ nanospheres and (**b**) nanorods in nanofluids.

As shown in Figure 3, the as-prepared nanorods have a small diameter of 4.6 ± 0.3 nm and a length of 22.0 ± 1.1 nm with a uniform particle size distribution. The nanospheres have a diameter of 4.3 ± 0.1 nm. After added to oils, nanospheres disperse uniformly, as shown in Figure 4a. Whereas, nanorods tend to contact each other, as shown in Figure 4b. Moreover, the size of the nanoparticles is observed to be increased. The nanorods have an average diameter of 6.8 nm and a length of 27.6 nm. And nanospheres with an average diameter of 7.6 nm are obtained. The reason for the increased particle size is that the exterior of the TiO_2_ nanoparticles as modified by oleic acid is a hydrophobic alkyl long chain with oil molecules coated on the surface of the TiO_2_ nanoparticles [23].

### 3.2. Breakdown Property

The positive breakdown properties of insulating oil and two types of TiO_2_ nanofluids are shown in Table 1. The breakdown voltages of TiO_2_ nanofluids are both higher than that of the insulating oil. The nanorods have the best performance in the improvement of the breakdown strength of the insulating oil. In the case of the addition of TiO_2_ nanorods, the breakdown voltage is increased by up to 55.5%. The improvement is more than twice that of nanospheres, which is only 25.9%. Of particular note, the time to breakdown is significantly increased by 83.2% with the presence of nanorods. According to the data on time to breakdown, it can be clearly seen that nanorods dramatically change the average propagation velocity of positive streamers, which is of great importance since it has a significant influence on the breakdown voltage of insulating liquid [24,25].

### 3.3. Pre-breakdown Streamer Propagation

**Figure 5:**
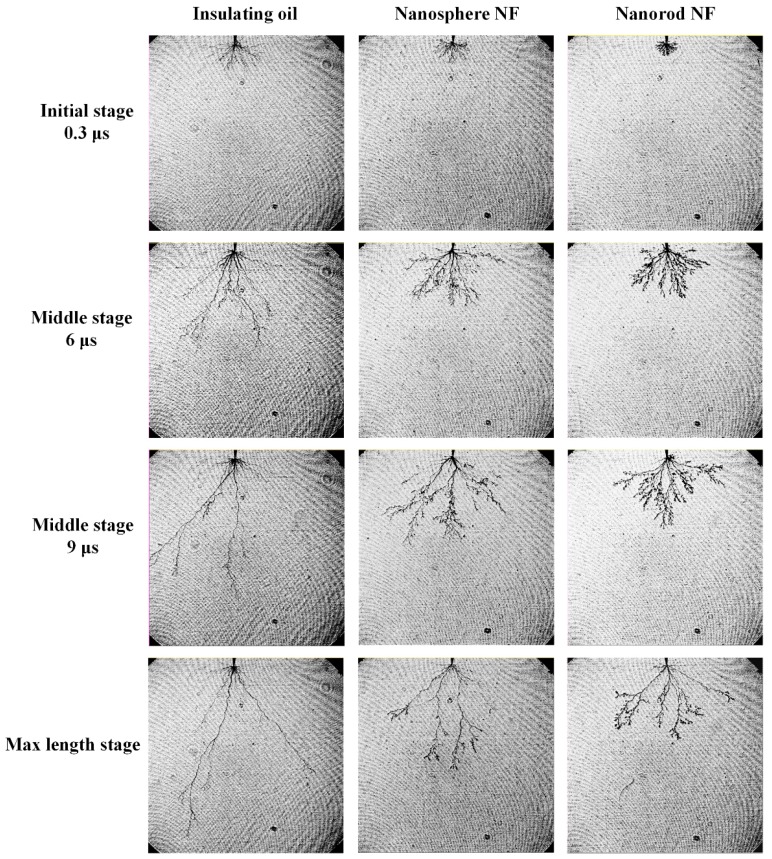
Pre-breakdown streamer propagation images for insulating oil, nanofluid with nanospheres and nanorods.

**Figure 6:**
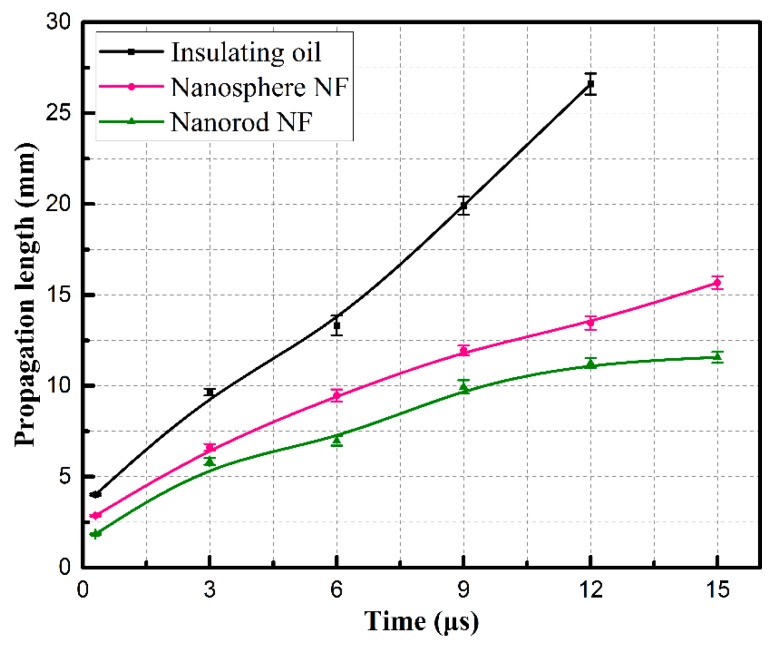
Positive streamer propagation length in insulating oil and nanofluids versus propagation times.

Figure 5 shows the shapes of pre-breakdown streamers from initiation to their maximum lengths (at 12 μs for insulating oil and 15 μs for nanofluids) in insulating oil and two types of nanofluids with nanospheres and nanorods, respectively. The streamers in insulating oil and nanofluids extend to the ground electrode once derived from the needle electrode. In insulating oil, pre-breakdown streamers show a tree-like shape, the same as those reported in References [26,27]. With the increase of propagation time, only two or three filaments are left to develop into main channels, the distance between which is obviously elongated. When compared with the streamer shapes in the insulating oil, streamers in nanofluids show a bush-like shape and have three considerable differences, especially for nanofluid with nanorods. First, more lateral branches are formed in nanofluid with nanorods than the fluid with nanospheres during the propagation process. Second, branches are much denser, and the distance between main channels is much closer in nanofluid with nanorods than that with nanospheres. Third, it is worth noting that streamer propagation length is kept even shorter in nanofluid with nanorods during the propagation process as presented in Figure 6. In addition, the average propagation velocity of streamers in insulating oil is 2.22 km/s according to the slope of curves in Figure 6, which is consistent with propagation velocity of second streamers in mineral insulating oil [6]. Whereas, in nanofluid with nanorods, the velocity is sharply decreased to 0.77 km/s, even lower than that of 1.04 km/s in nanofluid with nanospheres. This observation indicates that the addition of nanoparticles in a rod-like shape has a more significantly inhibiting effect on streamer propagation process than sphere-like ones.

### 3.4. Discussion

**Figure 7:**
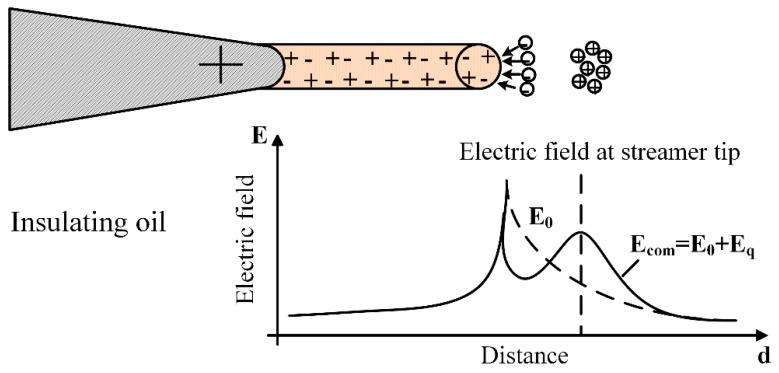
The electric field distribution in insulating oil ($E_{0}$: applied electric field, $E_{q}$: space–charge electric field).

In dielectric liquids, the breakdown strength is related to the streamer propagation, which is strongly affected by the electric field [3,5,6]. The electrons and positive ions are generated at streamer tips by ionization. When compared with positive ions, electrons move out of the ionization region with a much faster speed, leaving positive ions to become the transient space charges, as presented in Figure 7. In insulating oil, positive ions tend to assemble locally due to the filament shape of streamers, resulting in a greater distortion of the electric field. Thus, the electric field towards the ground electrode is enhanced by the space–charge field created by the positive ions, making it easier for streamers to propagate.

The greater inhibition on the propagation process of pre-breakdown streamers as well as the improvement on breakdown properties contribute to the difference of nanoparticle morphology. Nanoparticles are considered to have a large area of interface in nanocomposites [28,29], so streamers easily contact nanoparticles [30,31]. Whereas, streamers cannot go through the nanoparticles but must go around them, leading to more branches. This is similar to the treeing propagation in polymer nanocomposites proved by the scanning electron micrograph [32,33]. When compared with nanospheres, the streamers are more likely to be blocked by nanorods due to their larger collision cross-section, resulting in more branches.

**Figure 8:**
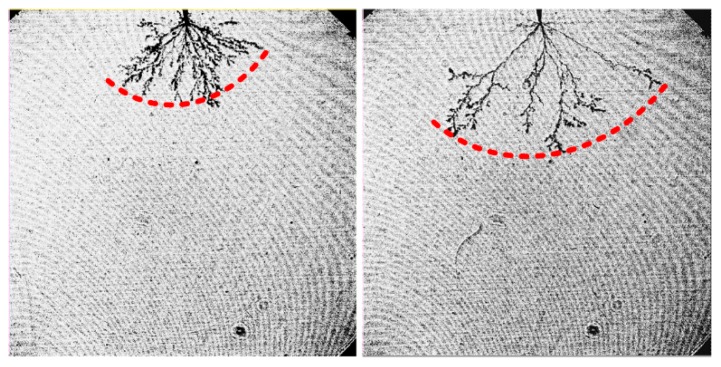
Streamers of nanofluid with nanorods.

**Figure 9:**
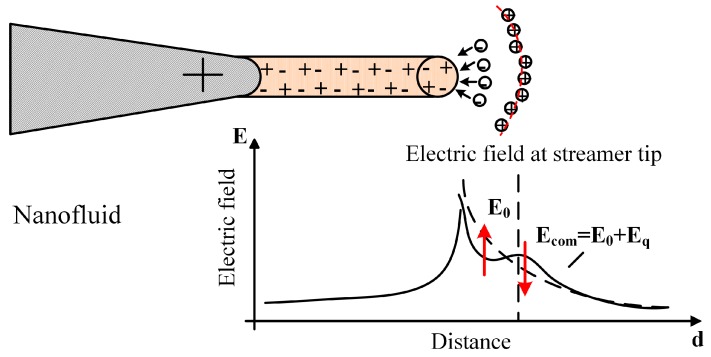
The electric field distribution in the nanofluid ($E_{0}$: applied electric field; $E_{q}$: space-charge electric field).

Furthermore, the large interfacial layer surrounding nanoparticles is beneficial for charge migration [30,31]. The interaction zone for charge migration is formed when interfacial layers overlap [31,32]. There is a higher probability for nanorods to come in contact with each other (as shown in Figure 4b), forming an extended interaction zone in which charges can move easily. This has also been reported in nanocomposites modified by TiO_2_ nanorods [34,35]. The promotion of charge migration in the nanofluid with nanorods is verified by the conductivity test according to the standard of IEC 60247-2004 as presented in Table 2. It should be mentioned that all observed results are mainly related to morphology, and do not consider the change of electron transport properties in the particles. Nanofluid with nanorods has a greater conductivity which means that charges migrate more easily. Some electrons may move in different directions through the interaction zone, further increasing the branches of streamers. In this case, streamer tips of nanofluids with nanorods distribute along an arc, as illustrated in Figure 8. So, the space charges generated at the streamer tips distribute more uniformly compared with that in the insulating oil or nanofluid with nanospheres, as presented in Figure 9. The distortion of the electric field is decreased, which considerably reduces the streamer propagation length. Therefore, the propagation of pre-breakdown streamers in nanofluid is greatly hindered by the presence of nanorods, resulting in the significant improvement in breakdown property. 

## 4. Conclusions

This paper investigated the effect of nanoparticle morphology on pre-breakdown and breakdown properties of insulating oil-based nanofluids, revealing the working mechanism for greater improvement by nanorods. The improvement of the breakdown property of nanofluid with nanorods is much larger than that of TiO_2_ nanospheres. Moreover, in nanofluid with TiO_2_ nanorods, pre-breakdown streamers exhibit a bush-like shape with the most branches and shortest propagation length compared with insulating oil and nanofluid with nanospheres. Thus, the addition of nanorods significantly suppresses the pre-breakdown streamer propagation, leading to a remarkable improvement in the breakdown property. Consequently, it is anticipated that TiO_2_ nanorods will provide a promising material for improving the dielectric strength of insulating oil and be highly desirable for future application in power equipment.

## Figures and Tables

**Table 1:** Positive breakdown properties of insulating oil and TiO_2_ nanofluids.

Sample	Breakdown Voltage (kV)	Standard Deviation (kV)	Time to Breakdown (μs)	Standard Deviation (μs)
Insulating oil	83.52	5.89	15.09	1.08
Nanofluid (Nanosphere)	105.19	5.76	22.38	1.98
Nanofluid (Nanorod)	129.85	2.54	27.65	1.27

**Table 2:** Conductivity of insulating oil and TiO_2_ nanofluids.

Sample	Insulating Oil	Nanofluid (Nanosphere)	Nanofluid (Nanorod)
**Conductivity (S/m)**	$6.4\times 10^{-13}$	$2.8\times 10^{-11}$	$4.5\times 10^{-11}$
